# You Are the Real Terrorist and We Are Just Your Puppet: Using Individual and Group Factors to Explain Indonesian Muslims’ Attributions of Causes of Terrorism

**DOI:** 10.5964/ejop.v12i1.1001

**Published:** 2016-02-29

**Authors:** Ali Mashuri, Lusy Asa Akhrani, Esti Zaduqisti

**Affiliations:** a Department of Psychology, University of Brawijaya, Malang, Indonesia; b Department of Islamic Education, STAIN Pekalongan, Pekalongan, Indonesia; University of Liverpool, Liverpool, United Kingdom

**Keywords:** Islamic fundamentalism, symbolic threat, realistic threat, attribution of terrorism, uncertainty avoidance, cognitive and emotional responses to mortality salience

## Abstract

The current study investigates the role of individual and intergroup factors in predicting Muslims’ tendency to attribute domestic terrorism in Indonesia to an external cause (i.e., The West) or an internal cause (i.e., radical Islamist groups). The results (N = 308) showed that intergroup factors of symbolic threat and realistic threat directly increased the external attribution and conversely decreased the internal attribution. Within the context of the current research, symbolic threat refers to Muslims’ perception that the norms and values of the West undermine Islamic identity. Realistic threat denotes Muslims’ perception that the economy and technology of the West undermine Islamic power. The individual factor of Islamic fundamentalism, which has to do with Muslims’ belief in the literal interpretation of and strict guidelines to Islamic doctrines, indirectly predicted both external attribution and internal attribution of terrorism as hypothesized, via the extent to which Muslims perceived the West as posing a symbolic threat, but not a realistic threat to Islamic existence. Uncertainty avoidance, a cultural dimension that describes the extent to which people view clear instructions as a pivotal source of concern to deal with societal problems, also significantly increased perceived symbolic threat and realistic threat, and this cultural dimension mediated the effect of Islamic fundamentalism on each of the intergroup threats. Finally, we found that the level of Islamic fundamentalism was dependent upon cognitive response, but not emotional response to mortality salience. The cognitive response to mortality salience denotes what Muslims are thinking about in coping with their own death whereas the emotional response denotes what Muslims are feeling about such issue. In particular, we found the cognitive response, but not the emotional response to mortality salience significantly gave rise to Muslims’ Islamic fundamentalism. These findings shed light on the importance of combining individual factors and group factors in explicating the dynamics of Muslims’ tendency to make attributions of causes of domestic terrorism. We discuss theoretical implications and study limitations, as well as practical actions policy makers could conduct to deal with Muslims’ Islamic fundamentalism and reduce the extent to which this particular group perceives the West as threatening their existence.

## Introduction

Extremism is not unique to a religion, but this phenomenon in Islam has drawn special attention given the growing acts of terrorism that continue to be employed by some Muslim radicals (Esposito & Mogahed, 2007). The most up-to-date notorious example involves terrorist acts by Islamic State (IS) in Syria and Iraq, which have spread to other countries, from Afghanistan, Egypt, and Libya to Algeria (Hoft, 2015). Terrorism is a complex and often unanticipated event, which makes it hard to comprehend immediately its nature and causes. People have a general tendency to seek explanations for such events by spontaneously connecting them to certain causes (Shaver, 2012). Within social psychology literature, this tendency is captured in the term “causal attribution”, which has been investigated by prior empirical studies to reveal what groups people blame and view as responsible for perpetrating Islamist terrorism. However, the approach of these studies tends to be particular in the sense that in explaining people’s attribution of terrorism, they distinguish between individual factors such as patriotism (Sahar, 2008) and emotions (Small, Lerner, & Fischhoff, 2006) and intergroup factors such as group membership (Doosje, Zebel, Scheermeijer, & Mathyi, 2007; Kimhi, Canetti-Nisim, & Hirschberger, 2009) or intergroup conflict and domination (Sidanius, Henry, Pratto, & Levin, 2004). Studies that integrate individual and intergroup factors to elucidate attributions of Islamist terrorism are therefore still lacking, which is the focal aim of our current paper.

We conducted this study in Indonesia, the largest Muslim population in the globe, which is home to an estimated number of 225,000,000 Muslims (Budiman, 2013). Over the past decade, terrorism has become one of the unresolved Indonesian problems (Arnaz & Marhaenjati, 2013). Among a series of terrorist attacks in this country, the Bali Bombings in 2002 were viewed as the most heinous ones (Karmini, 2012). Indonesian authorities have made much effort to crack down on terrorist groups by detaining and even killing some members and suspected members of these groups, as well as disseminating information that these radical groups are the real perpetrators of the terrorism (Perdani & Parlina, 2014). However, it is believed that much of the Indonesian public has disregarded such official reports and even otherwise externalizes the problem by blaming the West as having created terrorism in Indonesia (Jones, 2009; Mashuri & Zaduqisti, 2014a, 2014b). The West in this discourse is believed to be the real terrorists whereas Indonesian radical Islamists are just their puppets (Hilmy, 2010). We demonstrate that the extent to which Muslims perceive the West as posing a symbolic threat as well as a realistic threat to Islamic existence is directly accountable for increasing their tendency to blame the West as a perpetrator of domestic terrorism (i.e., external attribution of terrorism) and conversely decreasing their tendency to blame home-grown radical Islamists as the perpetrator (i.e., internal attribution of terrorism). We also show that either uncertainty avoidance or Islamic fundamentalism directly predicts symbolic threat, whereas the first but not the latter directly predicts realistic threat. Moreover, as it will become clear in the following, cognitive response but not emotional response to Muslims’ own death (i.e., mortality salience) is another individual factor that increases Islamic fundamentalism.

### Attributions of Cause of Terrorism as Predicted by Intergroup Threats

Early social psychologists (Heider, 1958; Kelley, 1967) assumed that the people commit attribution because of their desire to find causes and explanations for daily events, in order to gain a sense of control and predictability over their environments. Other theorists (e.g., Bohner, Bless, Schwarz, & Strack, 1988; Olson, Roese, & Zanna, 1996) expanded this theory by positing that threatening events are most likely to trigger attribution. Stephan, Ybarra, and Morrison (2009) theorized that within the intergroup relation context, people can feel threatened by two types of threats: symbolic and realistic threats. Symbolic threat refers to a threat emanating from ingroup members’ perception that the norms, values, or cultures of their group are being undermined by those of the outgroup members. Some Muslims indeed view the West as symbolically threatening Islamic existence because the current globalization underpinning the West’s dominance has brought new norms and values, which are to some extent in conflict with and even undermine traditional Islamic ways of life (Moghaddam, 2005). Realistic threat has to do with a threat emanating from ingroup members’ perception that the economy, power, or security of their group has been challenged by those of the outgroup. The Western supremacy in areas such as economy, technology, and politics has intensified Muslims’ perception that the power of Islam is under threat, which reflects how Muslims indeed subjectively view the West as posing a realistic threat to Islamic existence (Fair & Shepherd, 2006).

Prior studies have examined intergroup threat and attribution, but their focus was not on how the first directly impacts on the latter. Rather, intergroup threat and attribution have been investigated in combination and found to interact to affect negative outgroup attitudes in terms of ethnic prejudice and anti-Semitism (Becker, Wagner, & Christ, 2011) or negative affect against the threatening group (Costarelli, 2007). In addition, Dietz-Uhler and Murrell (1998) found that identity threat did not significantly affect people’s internal and external attributions in such a way that people reported an equal level of the attributions under threatening condition and non-threatening condition, more particularly when they strongly identified with their own group.

Unlike those studies, we proposed a new venue of research where intergroup threat is predicted as a potent, direct determinant of attribution. We built our arguments upon the model of ethnocentric attribution of bias (Weber, 1994). This model postulates that to protect their group esteem, ingroup members externally attribute their internal problems in such a way that they blame the outgroup rather than their group as responsible for their own problems. In contrast, to enhance their group esteem, ingroup members defensively deny their own group’s accountability for their own problems. These attributions, as found in the research by Kenworthy and Miller (2002), are in part fuelled by the perception of ingroup members that the outgroup poses a threat to their existence. Stated another way, when people perceive outgroup as threatening their own group, they tend to blame the outgroup rather than the ingroup for causing their own problems. Taken together, drawing on the model of ethnocentric attribution of bias, Muslims’ perception of the West as symbolic and realistic threats to Islamic existence may result in two opposing consequences: (1) an increased external attribution of the cause of terrorism, in which the West is blamed as causing terrorism and conversely, (2) a decreased internal attribution of terrorism, in which the radical Islamists are the ones to blame.

### The Impact of Islamic Fundamentalism on Intergroup Threats

Previous studies have examined the link between intergroup threat perceptions and Islamic fundamentalism. Altemeyer and Hunsberger (1992, p. 118) defined religious fundamentalism as “the belief that there is one set of religious teachings that clearly contains the fundamental, basic intrinsic, essential, inerrant truth about humanity and deity”. This widely cited definition of religious fundamentalism, however, should be interpreted with caution. This is because, as pointed out by Williamson, Hood, Ahmad, Sadiq, and Hill (2010), such definition originally derives from a particular historical context of Protestant movements in America. As a result, whether such definition can generalize into fundamentalism across major religions other than Christianity such as Buddhism, Hindu, or Islam is still debatable (Hood, Hill, & Williamson, 2005). Despite this controversy, scholars however converge towards the notion that what fundamentalism across those major religions have in common is resistance against alternative worldviews, intolerance of ambiguity, and narrow-mindedness (Moghaddam, 2008). With these characteristics, religious fundamentalism in general causes outgroup negativity including prejudice (Johnson, Rowatt, & LaBouff, 2010), hostility (Rothschild, Abdollahi, & Pyszczynski, 2009), and discrimination (Kirkpatrick, 1993). Drawing on these findings, we posited that the impact of religious fundamentalism in fostering people’s negative attitudes against the outgroup translates into the perception that this outgroup threatens these people’s own group.

In the studies by Jackson and Esses (1997), participants self-reporting as a Protestant, Catholic, Jew, and others (e.g., Muslim, Hindu) who were high in religious fundamentalism perceived single mothers and homosexuals, but not native Canadians and students as a symbolic threat, and this threat in turn prompted those individuals to hold the two groups to be responsible for their own social problems. High religious fundamentalists in this regard view the groups as symbolically threatening because mothers and homosexuals, but not native Canadians and students are considered to have norms and values that are starkly different from their standard religious norms and values. Duckitt (2006) found that among undergraduate students at Auckland University, New Zealand, a trait closely overlapping with religious fundamentalism termed right wing authoritarianism, which is characterized by conventionalism, authoritarian aggression, and authoritarian submission, gave rise to the perception that the threatening outgroups (i.e., drug dealers, feminists) posed a symbolic threat (i.e., threat to important norms, values, and traditions), but not a realistic threat (i.e., threat to social stability, security, and control) to the ingroup. In a similar vein, Mashuri, Zaduqisti, Sakdiah, and Sukmawati (2015) recently found that within the Indonesian context, Muslims’ Islamic fundamentalism positively related to the extent to which this religious group perceived the West as a symbolic threat (i.e., threat to Islamic traditions and culture), but not a realistic threat to Islamic existence (i.e., threat to Muslims’ economic and political power). All of these findings suggest that the more fundamentalist Muslims are, the more likely it is that they perceive the West as posing a symbolic but not a realistic threat to Islamic existence. This is because Islamic fundamentalists are characterized by a desire to hold traditional norms and values, thereby prompting Muslims high in Islamic fundamentalism are more sensitive and curious to symbolic rather than realistic threat in their relations with the West (Moghaddam, 2009, 2012). In a similar vein, Monroe and Kreidie (1997) stipulated that religious fundamentalism in general has to do more with identity crisis rather than political and economic crises. In particular, identity crisis denotes “a crisis of identity by those who fear extinction as a people or their absorption into an overarching culture to such a degree that their distinctiveness is undermined in the rush to homogeneity” (Marty & Appleby, 1991).

### The Mediating Role of Uncertainty Avoidance

Integrated threat theory (ITT) describes that cultural dimension of uncertainty avoidance serves one of the antecedents of intergroup threat perceptions (Stephan et al., 2009). As Hofstede (1991, p. 113) put it, people high in uncertainty avoidance “feel threatened by uncertain or unknown situations”. High uncertainty avoidance thus reasonably leads people to be susceptible to seeing an unfamiliar outgroup with different values, norms, and cultures as threatening (Stephan et al., 2009). High uncertainty avoidance also ignites the heightened need for security, which makes people high on this cultural dimension to be prone to experience realistic threat (Stephan et al., 2009). Taken together, drawing on the rationales of the ITT, uncertainty avoidance may augment both symbolic threat and realistic threat.

Furthermore, some studies have suggested that the extent to which people show high uncertainty avoidance depends on their level of religious fundamentalism. Compared to less religious fundamentalists, high religious fundamentalists are more resistant to ambiguity (Budner, 1962), more intolerant of inconsistency (Feather, 1964), and reported an augmented need for closure (Saroglou, 2002). Brandt and Reyna (2010) also found that religious fundamentalism significantly resulted in a greater preference for order and predictability, decisiveness, and discomfort with ambiguity, a cluster of psychological expressions of need for closure. As concluded by these authors, religious fundamentalism serves to attenuate instability and maintain certainty. Deriving from these rational and empirical findings, we contend that Muslims’ Islamic fundamentalism may increase uncertainty avoidance. This cultural dimension in turn makes Muslims perceive the West as posing an intergroup threat to Islamic existence. Uncertainty avoidance, therefore, is expected to mediate the effect of Muslims’ Islamic fundamentalism on symbolic and realistic threat perceptions towards the West.

### Mortality Salience and Islamic Fundamentalism

The mortality salience hypothesis within the terror management theory (TMT) posits that belief in the cultural worldviews functions to protect people from anxiety about their own death (Pyszczynski, Solomon, & Greenberg, 2003). As a consequence, reminders over their own death (i.e., mortality salience) should boost people’s need for the belief. As pointed-out by Salzman (2008), religious fundamentalism stands for the belief in the cultural worldviews that provides people with a defensive system to cope with the death-related anxiety. This implies that mortality salience and religious fundamentalism are two concepts that are presumably interconnected. Nevertheless, previous studies have escaped their attention to investigate the nature of the connection between the two, in order to verify which one really has a causal effect on another. Rather, existing studies are more interested in examining the combined effect of mortality salience and religious fundamentalism in molding a host of attitudes and behaviors such as hostility towards outgroups (Rothschild et al., 2009) and refusal to medical treatment (Vess, Arndt, Cox, Routledge, & Goldenberg, 2009).

Albeit not yet empirically investigated, McGregor (2006) argued that mortality salience is a potent antecedent of religious fundamentalism. In particular, this scholar reasoned that mortality salience is one of anxiety provoking threats. To alleviate these threats, people respond to mortality salience with defensively zealous reactions characterized by, inter alia, value adherence and close-mindedness that mark religious fundamentalism (Galen, 2011). In support of these arguments, a study conducted by Gailliot, Stillman, Schmeichel, Maner, and Plant (2008) revealed that mortality salience increased people’s adherence to salient norms and values.

Previous studies (Hunsberger, Alisat, Pancer, & Pratt, 1996; Hunsberger, Pratt, & Pancer, 1994) have revealed that people high in religious fundamentalism tended to think more about simple topics surrounding existential threats such as abortion and the death of the beloved one and demonstrated the same level of such cognitive complexity when persuaded to think about non-existential issues such as environmental dilemma. Other studies demonstrate that more religious people are the greater they have positive emotions (Christopher, Drummond, Jones, Marek, & Therriault, 2006) and the lesser they are anxious about death (Cohen et al., 2005). Corroborating these findings, Friedman (2008) found that cognitive response to people’s own death (i.e., mortality salience), which particularly has to do with simple religion-related concepts such as hell and heaven, God, sin, physical decay and so forth, significantly increased religious fundamentalism. In contrast, negative emotions in response to people’s own death such as anxiety, depression, sadness, loneliness, and fear did not significantly predict religious fundamentalism.

### Overview and Hypotheses of the Study

We operationalized the term “the West” in this study as the United States and its supposed allies, more particularly Western European countries and Israel. This operationalization built on some studies among Indonesian Muslims (Khisbiyah, 2009; Muluk, Sumaktoyo, & Ruth, 2013; Putra & Sukabdi, 2014; Siegel, 2000; Suciu, 2008; van Bruinessen, 2002) revealing that members of this religious group have pervasive negative sentiments against these countries believed as having conspired to debilitate Islam or Muslims. In case of Israel, these studies uncovered that some Indonesian Muslims’ hostility against this country is rooted at their unilateral claim that the U.S. has incessantly supported Israel’s occupation of Palestine. We accordingly provided participants with information on such operationalization at the beginning of the questionnaire.

We recruited only Indonesian Sunni Muslims as participants in the current research. Sunni in Indonesia is a dominant Islamic denomination that makes up 99% out of 225 million Muslims, whereas the rest are Shiites and Ahmadis constituting the Islamic minority groups (Mashuri & Zaduqisti, 2014c). While continuously persecuting minorities of Shiites and Ahmadis (Amirio, 2015; Fachrudin, 2015), some Sunni Muslims in Indonesia have demonstrated a growing trend of complete anti-West sentiments (Bayuni, 2015). These sentiments typically take form in call for the enforcement of Islamic law to displace democracy they harshly criticize as the representation of the Western liberal ruling system that is not aligned with the Islamic norms and values (Lestari & Seiff, 2015). Within the Indonesian context, there is not yet available information regarding how Shiites and Ahmadis view the West and whether these Islamic minorities equally demonstrate such negative sentiments. Building on these contextual backgrounds, the inclusion criterion of participants in the current research focused on Sunni Muslims.

We administered the questionnaire compiling all materials in the current research in Indonesian, following the standard procedure of a backward-forward translation (de Groot, Dannenburg, & van Hell, 1994). Nevertheless, an exception was made for scales adopted from the previous studies that were originally administered in Indonesian. The first author initially translated the original English scales into Indonesian. An independent bilingual proficient in both English and Indonesian then translated back the scales into English. In the third step, the first author compared those two English versions of the scales in an attempt of checking language biases. Finally, we refined the Indonesian version of the scales based on the existing language biases.

Drawing on theoretical rationales and empirical findings discussed above, we generated several hypotheses. The model of ethnocentric attribution of bias (Weber, 1994) suggests that with the aim of protecting their collective self-esteem, ingroup members attribute their own problems to outgroup (i.e., an external cause) rather than ingroup (i.e., an internal cause). People perform such attribution more strongly when they view outgroup as threatening their existence (Kenworthy & Miller, 2002). Drawing on these rationales, we predicted that both symbolic threat and realistic threat would increase external attribution of terrorism but decrease internal attribution of terrorism (*Hypothesis 1*). People high in Islamic fundamentalism are concerned more about maintaining traditional norms and values rather than power or economic status (Moghaddam, 2009, 2012; Monroe & Kreidie, 1997), which render them to become more sensitive to symbolic threat instead of realistic threat perceptions. From this argumentation, we predicted that Islamic fundamentalism would increase symbolic threat but not realistic threat (*Hypothesis 2*), wherein symbolic threat but not realistic threat would mediate the effect of Islamic fundamentalism on external attribution of terrorism (*Hypothesis 3a*) and internal attribution of terrorism (*Hypothesis 3b*).

According to integrated threat theory, uncertainty avoidance can be a potential precursor of both symbolic threat and realistic threat (Stephan et al., 2009). This cultural dimension may predict symbolic threat because people high in uncertainty avoidance are intolerant against different norms, culture, or values, which ultimately leads high fundamentalists to see them as a threat to their identity. Uncertainty avoidance may predict realistic threat because people high in this cultural dimension tend to demonstrate high need for security, which makes them more competitive oriented and accordingly view other groups as a threat to their political or economic standings. Building on these arguments, we subsequently predicted that uncertainty avoidance would increase both symbolic threat and realistic threat (*Hypothesis 4*). People high in religious fundamentalism have reported several heightened needs as earmarks of people’s tendency to avoid uncertainty such as predictability, order, and decisiveness (Brandt & Reyna, 2010). This suggests that religious fundamentalism may increase uncertainty avoidance. In light of these argumentations, we predicted that Islamic fundamentalism would increase uncertainty avoidance and in turn, this cultural dimension would increase symbolic threat (*Hypothesis 5a*) and realistic threat (*Hypothesis 5b*). These hypotheses denote that the effect of Islamic fundamentalism on symbolic threat and realistic threat is mediated by uncertainty avoidance. Finally, with reference to the findings in Friedman’s (2008) study, we predicted that cognitive response and not negative emotional response to mortality salience would increase Islamic fundamentalism (*Hypothesis 6*).

## Method

### Participants and Design

Of the 308 participants, 181 (58.8%) were undergraduate students from STAIN Pekalongan and 127 (41.2%) were undergraduate students from University of Brawijaya, both of which were in Java, Indonesia (210 were female, 93 were male, 5 did not mention their gender; *M*_age_ = 19.20; *SD*_age_ = 2.01). All participants self-reported as a Sunni Muslim. Most of the participants (280) were ethnically Javanese, the rest (19) were non-Javanese, and 9 did not indicate their ethnicity. Participants took part in this study voluntarily, in return of no rewards. By means of convenience sampling, we recruited participants on the basis of their willingness to take part in the current study and available access to approach them (Creswell, 2013). We designed this study as a correlational survey, wherein unless otherwise indicated, all variables were quantitatively measured using a scale. Our decision to choose quantitative design over qualitative design in the current study built upon one main consideration, taking into account arguments made by Creswell (2013). In particular, we aimed to approach systematically the problems in the current study pertaining to individual and group factors that contribute to Muslims’ tendency to attribute terrorist attacks to an internal cause or external cause. By doing so, we expected to obtain comparable data, make possible generalization to the wider population, and test the theories with hypotheses as elaborated above.

### Procedure and Measures

The administration of this study was in a classroom, in which participants were handed a questionnaire compiling the relevant scales and other research materials. We created the scales by averaging the items on which participants were asked to indicate their agreement on a 7-point *Likert* scale ranging between 1 (*not at all*) and 7 (*very much*). A recent study led by Dawes (2008) demonstrated no significant impact of the three different formats of 5-point, 7-point, and 10-point *Likert* scales in affecting mean variation, skewness, and kurtosis of the data. However, we decided to use 7-point instead of 5-point or 10-point *Likert* scales because psychometric literature (Nunnally & Bernstein, 1994) suggests that the first than the second and the third is more warranted to reach a good balance between having a measure with enough points of discrimination and having a measure without excessive response options.

At the beginning of the questionnaire, participants were provided with informed consent on which they were asked to affix signature upon their agreement to take part in this study. The next parts of the questionnaire were a series of items to assess each of the variables including emotional and cognitive response to mortality salience, Islamic fundamentalism, uncertainty avoidance, symbolic threat, realistic threat, external attribution of terrorism, and internal attribution of terrorism, respectively. *Responses to mortality salience* were assessed with the procedure modified from the study conducted by Greenberg, Porteus, Simon, Pyszczynski, and Solomon (1995) and by Friedman (2008). First, the mortality salience was induced by providing participants with the instructions following a standard procedure by Greenberg et al. (1995): “Please imagine that your own death arouses in you” and “To what extent do you agree that as you physically die and once you are physically dead, you will feel emotions and think about the topics as the following?” Then, participants were asked to rate the extent to which they will feel negative emotions such as sadness and fear and think of topics such as God, religion, and physical decay. The themes of these emotions and topics built upon the empirical findings in the study led by Friedman (2008) pertaining to the quantitative text analysis on how people respond to mortality salience. Overall, items to assess negative emotional responses were reliable (α = .79), so were those to assess cognitive responses (α = .78). *Islamic fundamentalism* was assessed with 20 items modified from the study by Altemeyer and Hunsberger (1992). This modification of this original scale was based on the study by Kunst, Thomsen, and Sam (2014), in which general terms were replaced by more specific ones, suited to Muslim fundamentalism. General terms such “God” were replaced with “Allah”, “sacred scripture” by “Qur’an”, “religion” by “Islam”. This modified scale (e.g., “Allah has given mankind a complete, unfailing guide to happiness and salvation, which must be totally followed) had an acceptable reliability (α = .68).

*Uncertainty avoidance* was assessed with five items adapted from the study conducted by Yoo, Donthu, and Lenartowicz (2011; e.g., “It is important to closely follow instructions and procedures”; α = .83). *Symbolic threat* was assessed with six items, with item number one to five being adopted from the study conducted by Mashuri, Zaduqisti, Sukmawati, Sakdiah, and Suharini (2015) and item number six was created by the authors in an exploratory way (e.g., “The possibility that the values ​​and culture of the West will continue to displace and weaken the values ​​and culture of Islam is something that deeply worries me”; α = .91). *Realistic threat* was assessed with six items adopted from the study conducted by Mashuri, Zaduqisti, Sukmawati, et al. (2015; e.g., “I fear that the strong power of the Western world will debilitate a bargaining position of the Islamic world in the global competition”; α = .92). *External attribution of terrorism* was assessed with six items (e.g., “The West is the true perpetrator of terrorism in Indonesia”; α = .79). *Internal attribution of terrorism* was assessed with four items (e.g., “Terrorism in Indonesia is due to the errors radical Muslim have done in interpreting Islam”; α = .61). Both of these scales were created by the authors in an exploratory way, to suit them to the Indonesian context. At the end of the questionnaire, participants were asked to indicate their age, gender, ethnicity, university affiliation, and Islamic denomination (i.e., Sunni, Shia, Ahmadiyya, and others). Upon finishing, participants were debriefed and thanked.

## Results

### Preliminary Analyses

#### Descriptive Statistics

Table 1 presented descriptive statistics and correlations among observed variables in the current study. Inspections of a one-sample *t*-test revealed that all variables were high as they are significantly above the midpoint of 4, except for internal attribution of terrorism that was conversely low as it is significantly below the midpoint of 4. Each variable tended to correlate with one another, except for emotional response to mortality salience that significantly correlated only with cognitive response to mortality salience.

**Table 1:** Correlations Among Observed Variables

Variable	*M*	*SD*	1	2	3	4	5	6	7	8
1. Emotional Response to Mortality Salience	4.85***	1.35	−	.21**	.04	.06	.03	.09	-.08	-.08
2. Cognitive Response to Mortality Salience	6.33***	.82		−	.31**	.29**	.19**	.15**	.09	-.13*
3. Islamic Fundamentalism	4.85***	.67			−	.35**	.30**	.21**	.09	-.03
4. Uncertainty Avoidance	6.22***	.83				−	.36**	.25**	.17**	-.17**
5. Symbolic Threat	5.75***	1.23					−	.50**	.31**	-.24**
6. Realistic Threat	5.32***	1.46						−	.33**	-.26**
7. External Attribution of Terrorism	4.51***	1.17							−	-.14*
8. Internal Attribution of Terrorism	2.73***	1.19								−

*p < .05. **p < .01. ***p < .001.

#### Item Parceling

Our focus in the current paper was on the relations among latent constructs instead of the relations among observed variables or items within these constructs. As such, we implemented item parceling to create indicators in our structural models (Little, Cunningham, Shahar, & Widaman, 2002). With such focus, item parceling is said to be less problematic and even warranted (Rogers & Schmitt, 2004). We transformed the observed variables into the latent variables by means of item-parceling. This procedure built upon the result of exploratory factor analysis (EFA) with an oblique rotation (i.e., PROMAX: Little et al., 2002; Hall, Snell, & Foust, 1999). If the EFA revealed that the variable is unidimensional, we used an item-balancing technique to construct the item parceling (Little et al., 2002; Sass & Smith, 2006). If the EFA revealed that the variable is multidimensional, we used a domain-representative technique (Kishton & Widaman, 1994) to construct the item parceling. Parcels may consist of unequal items. Under this condition, the item-balancing technique can still maintain parcels with balanced factor loadings. However, unequal items can make one or more parcels no longer domain representative as these parcels do not cover items from all dimensions or facets (Coffman & MacCallum, 2005).

We analyzed the data by means of Mplus version 6 (Muthén & Muthén, 1998-2010). The data contained some missing values as one or more items were not completed by the participants. However, the missing values were only .92%, which is considered as very small and thereby inconsequential to bias the statistical analysis as they are far less than 5% (Schafer, 1999). The Mplus also revealed that the data contradicted the assumption of multivariate normality (*Skewness*: 84.53, *M* = 40.57, *SD* = 1.47, *p* < .001; *Kurtosis*: 619.44, *M* = 524.51, *SD* = 3.64, *p* < .001). Based on these results, we decided to use MLR as an appropriate estimator for data that violate multivariate normality and that contain missing values (Wang & Wang, 2012).

#### Assessment of Construct Validity

Prior to hypotheses testing, assessment of the construct validity of the measurement model is highly recommended (Anderson & Gerbing, 1988; MacCallum, 1986). This assessment could take form in both convergent validity and discriminant validity, using confirmatory factor analysis (CFA: Brown, 2006; Harrington, 2008). Convergent validity connotes the extent to which theoretically overlapping indicators or constructs are highly correlated, whereas discriminant validity connotes the extent to which theoretically different indicators or constructs are not strongly interrelated. Convergent and discriminant validities of the measurement model hold when CFA results in good fits to the data. The goodness of fits of the measurement model was assessed with various parameters including the relative chi-square (i.e., the value of chi-square divided by the degree of freedom), Root Mean Square Error of Approximation (RMSEA), Comparative Fit Index (CFI), and Tucker-Lewis Index (TLI). Following recommendations (Hu & Bentler, 1999; Kline, 1998), the measurement model is said to live up to very good fits to the data if the relative chi-square is less than 2 or 3, whereas RMSEA should be less than .05 and CFI and TLI should be more than .95. As shown in Table 2, the hypothesized eight-factor oblique, which allowed all latent constructs or factors to correlate one another, resulted in very good fits to the data than either the eight-factor orthogonal, which allowed all latent constructs to be independent of one another, ∆χ^2^ (35) = 321.47, *p* < .001, or one factor, which specified all item parcels or indicators to load to a single latent construct, ∆χ^2^ (29) = 1449.23, *p* < .001. These findings overall confirmed that item parcels that serve as indicators in the hypothesized measurement model strongly loaded to their respective latent constructs (i.e., convergent validity), but weakly loaded to other latent constructs (i.e., divergent validity). ^i^

**Table 2:** Comparison of Fit Indices of the Hypothesized Measurement Model (Eight-Factor Oblique), the Second Measurement Model (Eight-Factor Orthogonal), and the Third Measurement Model (One Factor)

Measurement model	Fit index					
	χ^2^	*df*	χ^2^/*df*	RMSEA	CFI	TLI
(1) Eight-factors oblique	268.05	181	1.48	.04 (90% CI [.03, .05])	.97	.96
(2) Eight-factors orthogonal	640.23	216	2.96	.08 (90% CI [.07, .09])	.84	.83
(3) One factor	2013.31	210	9.59	.17 (90% CI [.16, .17])	.31	.25

#### Assessment of Item Parceling Model

When a construct is measured by a scale consisting of multiple items, indicators within this construct could be derived from three different aggregation levels: total disaggregation, partial disaggregation, and total aggregation (Coffman & MacCallum, 2005). Total disaggregation indicates that each item functions as an indicator for a construct. Partial disaggregation is characterized by several items that are averaged or summed to create parcels as an indicator for a construct. In a totally aggregated model, all items within a scale are averaged or summed in such a way that a construct represents an observed variable rather than a latent variable.

Some studies have found that the use of partial disaggregation or item parceling model is more advantageous than total disaggregation model in the sense that the first more than the latter resulted in less biased parameter estimates and better overall goodness of fits to the data, either in real or simulation studies (Bandalos, 2002). Compared to a total aggregation model, both total disaggregation model and partial disaggregation model are more advantageous because they can take into account error variances, which are undeniable in any psychological measurements. As such, in a situation where a construct is assessed with multiple items, the use of a total disaggregation model or a partial disaggregation model is more recommended than a total aggregation model that unrealistically assumes no measurement errors (Kline, 1998).

Table 3 presented comparison of the three aggregation models discussed above. As shown in this table, the partial disaggregation model as the hypothesized structural model in the current study turned out to have better overall goodness of fits to the data vis-à-vis a total disaggregation model and a total aggregation model. These results suggested that the partial disaggregation model more than the total disaggregation model and the total aggregation model is more reliable for use to test the hypotheses in the current study.

**Table 3:** Comparison of Fit Indices of the Total Disaggregation Model, Partial Disaggregation Model, and Total Aggregation Model

Structural model	Fit index					
	χ^2^	*df*	χ^2^/*df*	RMSEA	CFI	TLI
1. Total disaggregation model	3089.63	1525	2.03	.06 (90% CI [.06, .061])	.76	.74
2. Partial disaggregation model	279.24	195	1.43	.04 (90% CI [.03, .05])	.97	.96
3. Total aggregation model	24.04	14	1.72	.05 (90% CI [.01, .08])	.96	.92

### Hypotheses Testing

To test the hypotheses, we implemented a full model of structural equation modeling (Kelloway, 1998). In particular, this full model built upon the partial disaggregation model discussed above. In this hypothesized model, we allowed variables within the same group of constructs to correlate (McGartland Rubio, Berg-Weger, & Tebb, 2001). Accordingly, emotional responses to mortality salience and cognitive responses to mortality salience were allowed to correlate, and so were symbolic threat and realistic threat as well as external attribution of terrorism and internal attribution of terrorism.

**Figure 1:**
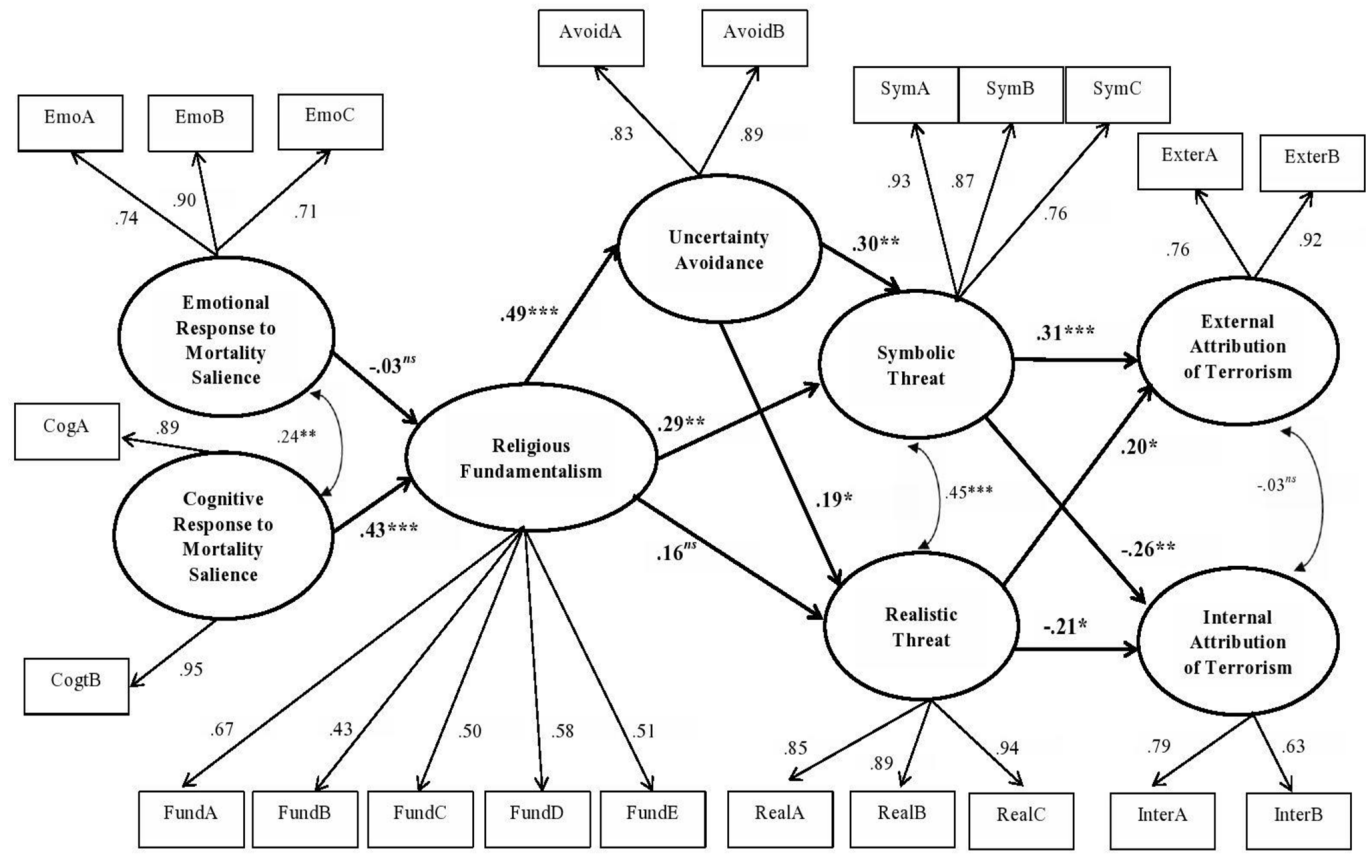
The hypothesized full model of the relationships among external attribution of terrorism, internal attribution of terrorism, symbolic threat, realistic threat, uncertainty avoidance, Islamic fundamentalism, cognitive response to mortality salience and emotional response to mortality salience. *Note*. The numbers were standardized path coefficients and factor-loadings. ExterA−ExterB = External Attribution of Terrorism Parcel A and Parcel B; InterA−InterB = Internal Attribution of Terrorism Parcel A and Parcel B; SymA = Symbolic Threat Parcel A; SymB = Symbolic Threat Parcel B; SymC = Symbolic Threat Parcel C; RealA = Realistic Threat Parcel A; RealB = Realistic Threat Parcel B; RealC = Realistic Threat Parcel C; AvoidA−AvoidB = Uncertainty Avoidance Parcel A and Parcel B; FundA−FundE = Islamic fundamentalism Parcel A, Parcel B, Parcel C, Parcel D, and Parcel E; EmotA−EmoC = Emotional Response to Mortality Salience Parcel A, Parcel B, and Parcel C; CogA and CogB = Cognitive Response to Morality Salience Parcel A and Parcel B. *^ns^p* ≥ .05 (not significant). *p < .05. **p < .01. ***p < .001. All factor-loadings were significant at *p* < .001.

Overall, the hypothesized model resulted in very good fits to the data, relative chi-square = 1.43, RMSEA = .037 (90% CI [.027, .047]), CFI = .97, TLI = .96. This model explained 17% variance of internal attribution of terrorism (*SE* = .05, *p* = .001), 20% variance of external attribution of terrorism (*SE* = .05, *p* < .001), 9% variance of realistic threat (*SE* = .04, *p* = .016), 26% variance of symbolic threat (*SE* = .07, *p* < .001), 24% variance of uncertainty avoidance (*SE* = .07, *p* = .001), and 18% variance of Islamic fundamentalism (*SE* = .07, *p* = .012). Figure 1 presented path coefficients and factor loadings of the hypothesized full model. As shown in Figure 1, symbolic threat significantly increased external attribution of terrorism (β = .31, 95% CI [.153, .456], *SE* = .08, *p* < .001, squared semi-partial correlation [sr^2^] = .08, power = .98), but decreased internal attribution of terrorism (β = -.26, 95% CI [-.424, -.086], *SE* = .09, *p* = .003, sr^2^ = .06, power = .84), and so did realistic threat—for the effect of realistic threat on external attribution of terrorism (β = .20, 95% CI [.039, .355], *SE* = .08, *p* = .015, sr^2^ = .03, power = .76); for the effect of realistic threat on internal attribution of terrorism (β = -.21, 95% CI [-.402, -.018], *SE* = .10, *p* = .032, sr^2^ = .04, power = .70).^ii^ These results substantiated *Hypothesis 1*. Islamic fundamentalism significantly increased symbolic threat (β = .29, 95% CI [.087, .494], *SE* = .10, *p* = .005, sr^2^ = .09, power = .95), but not realistic threat (β = .16, 95% CI [-.022, .348], *SE* = .09, *p* = .083), in support of *Hypothesis 2*. In line with Hypothesis 3a, the effect of Islamic fundamentalism on external attribution of terrorism was significantly mediated by symbolic threat (β = .09, 95% CI [.007, .171], *SE* = .04, *p* = .032, kappa squared [k^2^] = .22, power = .84) and not realistic threat (β = .03, 95% CI [-.012, .076], *SE* = .02, *p* = .149).^iii^ Symbolic threat but not realistic threat also significantly mediated the effect of Islamic fundamentalism on internal attribution of terrorism—for the indirect effect of symbolic threat, (β = -.07, 95% CI [-.144, -.005], *SE* = .04, *p* = .036, k^2^ = .22, power = .64); for the indirect effect of realistic threat, (β = −.03, 95% CI [-.086, .018], *SE* = .03, *p* = .197). These findings were in line with *Hypothesis 3b*.

Uncertainty avoidance turned out to significantly increase both symbolic threat (β = .30, 95% CI [.126, .481], *SE* = .09, *p* = .001, sr^2^= .05, power = .98) and realistic threat (β = .19, 95% CI [.030, .343], *SE* = .08, *p* = .019, sr^2^ = .02, power = .67), as predicted in *Hypothesis 4*. This cultural dimension also significantly mediated the effect of Islamic fundamentalism on symbolic threat (β = .15, 95% CI [.046, .249], *SE* = .05, *p* = .004, k^2^ = .16, power = .98) and realistic threat (β = .09, 95% CI [.009, .173], *SE* = .04, *p* = .03, k^2^ = .09, power = .65), corroborating *Hypothesis 5a* and *Hypothesis 5b*. Finally, we found in line with *Hypothesis 6* that cognitive response to mortality salience significantly increased Islamic fundamentalism (β = .43, 95% CI [.265, .599], *SE* = .09, *p* = < .001, sr^2^ = .25, power = 1.00), but emotional response did not too (β = −.03, 95% CI [−.168, .113], *SE* = .07, *p* = 698).

### Alternative Models

Some alternative models were examined to verify the plausibility of the hypothesized model. Comparison of nested models was tested on the basis of chi-square difference test, whereas Akaike Information Criteria (AIC) and Bayesian Information Criteria (BIC) were employed for comparison of non-nested models. A model with lower AIC and BIC indicates a better fit to the data (Nylund, Asparouhov, & Muthén, 2007). To be concluded as significant, the difference of AIC and BIC between two models should be at least 4 points (Burnham & Anderson, 2004). ^iv^

#### Alternative Model 1

**Figure A.2:**
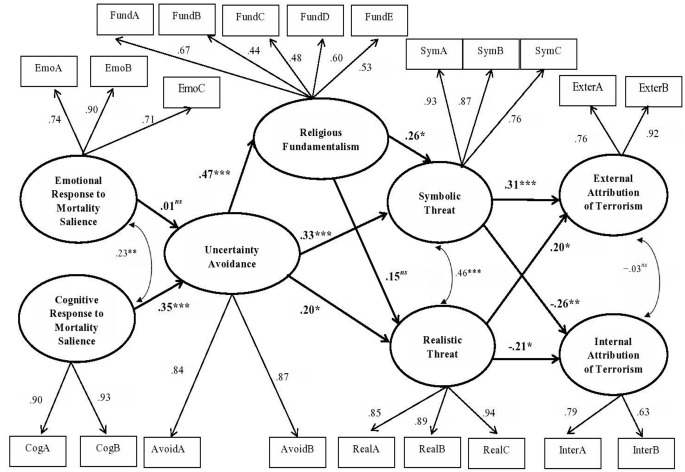
The Alternative Model 1 in which uncertainty avoidance is the antecedent of religious fundamentalism. *Note*. The numbers were standardized path coefficients and factor-loadings. ExterA−ExterB = External Attribution of Terrorism Parcel A and Parcel B; InterA−InterB = Internal Attribution of Terrorism Parcel A and Parcel B; SymA−SymC = Symbolic Threat Parcel A, parcel B, and Parcel C; RealA−RealC = Realistic Threat Parcel A, parcel B, and Parcel C; AvoidA−AvoidB = Uncertainty Avoidance Parcel A and Parcel B; FundA−FundE = Religious Fundamentalism Parcel A, Parcel B, Parcel C, Parcel D, and Parcel E; EmoA−EmoC = Emotional Response to Mortality Salience Parcel A, Parcel B, and Parcel C. CogA-CogB = Cognitive Response to Mortality Salience Parcel A and Parcel B. *^ns^p* ≥ .05 (not significant). *p < .05; **p < .01; ***p < .001. All factor-loadings were significant at *p* < .001.

The first alternative model was a non-nested model, in which uncertainty avoidance was specified as preceding Islamic fundamentalism (see Figure A.2 in Appendix 2). Uncertainty may directly lead to Islamic fundamentalism given that heightened identification with groups, more typically those with high entitativity such as religious groups, serves as a defensive mechanism to mitigate the uncertainty (Hogg, Adelman, & Blagg, 2010). The AIC (19009.25) and BIC (19307.66) of this alternative model were significantly greater than the hypothesized model (AIC = 18999.52; BIC = 19297.93), ∆AIC = .9.73, ∆BIC = 9.73.

#### Alternative Model 2

**Figure A.3:**
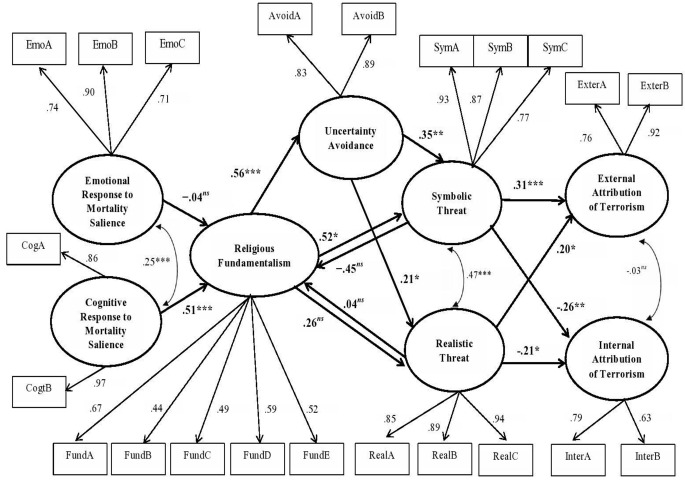
The Alternative Model 2 by assuming the bidirectional relationships among religious fundamentalism, symbolic threat, and realistic threat. *Note.* The numbers were standardized path coefficients and factor-loadings. ExterA−ExterB = External Attribution of Terrorism Parcel A and Parcel B; InterA−InterB = Internal Attribution of Terrorism Parcel A and Parcel B; SymA−SymC = Symbolic Threat Parcel A, parcel B, and Parcel C; RealA−RealC = Realistic Threat Parcel A, parcel B, and Parcel C; AvoidA−AvoidB = Uncertainty Avoidance Parcel A and Parcel B; FundA−FundE = Religious Fundamentalism Parcel A, Parcel B, Parcel C, Parcel D, and Parcel E; EmoA−EmoC = Emotional Response to Mortality Salience Parcel A, Parcel B, and Parcel C. CogA-CogB = Cognitive Response to Mortality Salience Parcel A and Parcel B. *^ns^p* ≥ .05 (not significant). *p < .05. **p < .01. ***p < .001. All factor-loadings were significant at *p* < .001.

The second alternative model was a nested model, in which the path from Islamic fundamentalism to symbolic threat and realistic threat was made bidirectional (see Figure A.3 in Appendix 2). Shaffer and Hastings (2007) found that participants exposed to threat demonstrated a higher degree of Islamic fundamentalism. In a more specific context, the political and cultural supremacies of the United States in particular and the West in general over Islamic countries in the current globalization era constitute a situational factor that has augmented Muslims’ Islamic fundamentalism and radicalism (Rogers et al., 2007). The chi-square of the hypothesized model (χ^2^ = 260.144, *df* = 195) was not significantly different from that of this alternative model (χ^2^ = 257.547, *df* = 193), ∆χ^2^ (2) = 2.60, *p* > .05.^v^

#### Alternative Model 3

The third alternative model was another nested model by adding the direct path from cognitive responses to mortality salience to symbolic threat and realistic threat (see Figure 4 in the Appendix). Greenberg et al. (1990) for example found that mortality salience directly gave rise to the ingroup’s negative evaluations of the threatening outgroup. The chi-square of the hypothesized model (χ^2^ = 260.144, *df* = 195) was not significantly different from this alternative model (χ^2^ = 250.412, *df* = 193), ∆χ^2^ (2) = .732, *p* > .05.

## Discussion

The current study aimed at simultaneously investigating individual factors and intergroup factors that affect the extent to which Indonesian Muslims perceive that terrorism in their country is caused externally by the West or internally by Islamist radicals. We found the concerted effect of symbolic threat and realistic threat in increasing external attribution of terrorism and, in contrast, decreasing internal attribution of terrorism. As predicted, Islamic fundamentalism resulted in greater symbolic threat, but not realistic threat. The former, but not the latter intergroup threat also turned out to mediate the effect of Islamic fundamentalism on either external or internal attributions of terrorism. We also found that uncertainty avoidance augmented both symbolic threat and realistic threat, and ultimately this cultural dimension mediated the effect of Islamic fundamentalism on each of these intergroup threats. Finally, cognitive instead of emotional response to mortality salience, as hypothesized, significantly prompted Muslim to be more religiously fundamentalist.

The finding in this study suggesting that symbolic threat and realistic threat substantially impacted on a greater external attribution of terrorism is in line with integrated threat theory (Stephan et al., 2009). As concluded in a meta-analysis study by Riek, Mania, and Gaertner (2006), both symbolic and realistic threats have been confirmed as triggering various negative outgroup attitudes including prejudice and discrimination. However, another finding in this study that symbolic threat and realistic threat have resulted in a lesser internal attribution of terrorism may develop and extend the ITT. This is because ITT has heavily focused on the effect of intergroup threats on negative outgroup attitudes, paying little attention to how these threats have empirical consequences on ingroup attitudes. However, Stephan et al. (2009) suggested that intergroup threats potentially contribute to more favorable ingroup attitudes. One of the findings in this study has substantiated this argumentation in which we observed that Muslims’ perception that the West has symbolically and realistically threatened Islamic existence led them to deny the Indonesian radical Islamists as being accountable for the domestic terrorism. To develop these findings and verify their consistency, future studies, however, require investigating terrorist events outside Indonesia, such as those in todays’ Syria and Iraq.

Existing studies regarding the effect of Islamic fundamentalism on negative intergroup attitudes and behaviors have reported mixed findings. As reviewed by Rothschild et al. (2009), fundamentalists across religions have supported or even actively taken part in international wars, terrorism, and violence. Fundamentalism has also been found to ignite prejudice, religious ethnocentrism, and support for militarism. On another hand, some studies have revealed that Islamic fundamentalism did not have a direct role in provoking such negativities, wherein such direct effect was moderated by some factors such as the belief in establishing Islam peacefully and justification of violent actions (e.g., Putra & Sukabdi, 2014), and compassionate values (Rothschild et al., 2009). The findings in this study thereby are parallel with the second line of studies, but with a different direction. More particularly, we found that the route from Islamic fundamentalism to the negativities in terms of external attribution of terrorism did not pass a direct, toll road, but an indirect one, via symbolic threat, but not realistic threat.

It is highly likely that the attributional effects as found in this study were not attributable to the specific characteristics of Islamic fundamentalism per say, but were attributable to the extent to which participants identified with the radical Islamists perpetrating the terrorist attacks. Stated another way, Islamic fundamentalism in our current study may have simply been a marker of the extent to which religious fundamentalists perceive the radical Islamists as ingroup members. This can be the case because people high in Islamic fundamentalism may view radical Islamists as ingroup members and accordingly, engage in self or group-serving attributions. People low in Islamic fundamentalism in contrast may not view the radical Islamists as ingroup members and thereby do not engage in the same defensive attributions. To verify this possibility, future studies need to assess the degree to which Muslims identify with the radical Islamists, which presumably moderate the degree to which Islamic fundamentalism predicts attributional biases.

In the current study we measured internal attribution of terrorism after external attribution of terrorism, which would be criticized as being potentially problematic. We indeed assumed in this study that blaming radical Islamists is conceptually the same as making internal attribution. Nevertheless, the perception that radical Islamists represent ingroup may be driven by the fact that participants first rated attributions to the West which could have shifted ingroup vis-à-vis outgroup boundaries such that radical Islamists, who may have otherwise been considered an “external attribution target” became an “internal attribution target”. However, we contend that Muslims are prone to categorize radical Islamists as their ingroup whereas the West as outgroup, despite the fact that they are very moderate and hence lowly identify with radical Islamists. The reason is that Muslims, more specifically Muslims in Indonesia, regardless of their religious orientations, are vulnerable to malevolent conspiracy theories portraying that the West is suspected of engineering and masterminding domestic or even international terrorism (Bowen, 2007; Jones, 2002, 2009; Mashuri & Zaduqisti, 2014a, 2014b; Perlez, 2002; Smith, 2005). This type of negative thinking in part stems from an array of perceived deprivations such as injustice, defeat, and powerlessness Muslims subjectively experience in their relations with the West (Suciu, 2008). People in general tend to feel these deprivations as an aversive experience that threaten their group esteem, whereas belief in conspiracy theory in this regard serves a defensive mechanism to alleviate this threat (Swami, 2012). We accordingly contend that owing to their proneness to such conspiracy theories, Muslims deny that radical Islamists are accountable for terrorism, even if they do not feel deeply attached to these extremist groups. To verify these argumentations, however, future studies can reverse the order of the attributional measures, in which internal attribution of terrorism is assessed prior to external attribution of terrorism.

Stephan et al. (2009) suggested that at a cultural level, intergroup threat perceptions are not only affected by how people respond to uncertainty (i.e., uncertainty avoidance) but also how they respond to power distribution and hierarchy within a society. This latter phenomenon is termed power distance as a cultural dimension that constitutes the extent to which people agree that power and social hierarchy within a society are distributed unequally (Hofstede, 1991). People high in power distance, therefore, highly support such social disparities. As argued by Stephan et al. (2009), given that cultures high in power distance are characterized with high indexes of aggression and conflict, the greater this cultural dimension is the greater are intergroup threat perceptions. Next studies, therefore, may also assess power distance along with uncertainty avoidance to look at their effects on intergroup threat perceptions.

The background of the current research was very particular, limited to the Indonesian context where Muslims constitute a religious majority group. Muslims in the Middle East and other countries in Asia such as Malaysia and Pakistan are equally the majority group. However, in other parts of the world such as Europe and America, Muslims are the minority group and this fact may eventually raise the question whether the findings of the current research could generalize into Muslims living in these regions. Nevertheless, Moghaddam (2006) as well as Krueger and Laitin (2008) argued that a major factor that ignites Muslims’ hostility across the globe against the West is perceived discrimination and injustice. Some studies have empirically verified this notion. Muslims as a minority group in Europe tend to perceive the West as posing a threat to their group existence because they feel that the West has discriminated and treated them unjustly (Hopkins, 2011). These perceived discrimination and injustice ultimately provoke identity conflict among Muslims in Europe, which is characterized by strong ingroup identification with Islam and weak national identification with the host country (Hopkins, 2011; Verkuyten & Yildiz, 2007). Combined together, such identity conflict has been confirmed to render Muslims in Europe to have negative attitudes against the West (Martinovic & Verkuyten, 2014; Verkuyten, 2007). In Indonesia, a recent study has also been in support of these findings in which among some Sunni Muslims, such perceived injustice augmented this religious group’s support for Islamic law (Muluk et al., 2013) and religious radicalism (Putra & Sukabdi, 2014) that all together denote anti-West sentiments. With reference to this identical pattern of empirical findings across a different context, we believe that the current research could be applied to Muslims living as a minority group in non-Muslim regions such Europe, America and so forth, which consequently helps broaden its readership.

A noteworthy limitation in the current study is that we did not measure participants’ group esteem as a Muslim after the attributions of terrorism. This procedure is potentially of high importance to verify the truism of the model of ethnocentric attribution of bias (Weber, 1994). As discussed earlier, this model posits that on one side, heightened external attribution serves a medium through which people protect their group esteem in dealing with the threatening outgroup. On the other side, when people encounter a problem that threatens their group, they respond to this problem by denying the responsibility on the part of their own group, in order to enhance their group esteem. Implicatively, group esteem as a Muslim assessed after the attributions of terrorism should positively correlate to external attribution of terrorism but negatively correlate to internal attribution of terrorism. Future studies could empirically examine these hypotheses. Another limitation that deserves discussing is that the effect of mortality salience on Islamic fundamentalism in the current student is not causative but correlational in nature as it was measured rather than manipulated. To overcome this drawback, next studies thus can create a non-mortality condition and then compare it to a mortality salience condition as examined in the current study. This procedure is very pivotal to establish the causative effect of mortality salience on Islamic fundamentalism and to assess whether the pattern of relationships among variables as found in the current study is stronger under mortality salience condition than non-mortality salience condition. We also acknowledge that convenience sampling that we used potentially limits the generalizability of the findings in the current study. To ameliorate this drawback, future studies may employ random sampling, in an attempt of getting more representative samples and extending the generalizability of the current research into much wider Muslim populations in Indonesia. Finally, we recommend that future studies employ qualitative approach to complement the current research. This approach may use an in-depth interview or a focus group discussion, especially to grasp what are the precise meanings of the West according to Muslims. We indeed constructed in the current research the meanings of the West as the U.S. and Western countries as well as Israel portrayed as it allies, with reference to reliable, empirical sources from existing studies among Muslims in Indonesia (Khisbiyah, 2009; Muluk et al., 2013; Putra & Sukabdi, 2014; Siegel, 2000; Suciu, 2008; van Bruinessen, 2002). However, qualitative research is still highly beneficial to cultivate the specific reasons why Muslims construe those countries as representing the West that are perceived as threatening Islamic existence. These reasons could enhance the objectivity of the meanings of the West within the context of the current research.

We propose two practical implications. First, as suggested by Rothschild et al. (2009), the religious community needs to accentuate the importance of compassionate values and teachings of love and acceptance, which are commonly shared by most religions. These authors found that inducing compassionate values among religious fundamentalists has attenuated their hostility against outgroups in dealing with mortality salience. The cultivation of compassionate values can be implemented through so-called omniculturalism (Moghaddam, 2012), a policy to promote human commonalities more than human differences in all levels of educational institutions. By internalizing human commonalities, children in particular and the public in general are taught about the importance of a superordinate identity that transcends social particularities in terms of ethnicity, gender, or religion. Omniculturalism in this manner can promote compassionate values of love and acceptance to others, and it is thereby said as a promising policy to reduce various ingroup extremism and ethnocentrism including Islamic fundamentalism (Moghaddam, 2012).

Second, intergroup threat perceptions can be reduced by enhancing intergroup contact, which is undeniable in the current globalization era. A meta-analysis by Pettigrew and Tropp (2008) has revealed that intergroup contact reduces intergroup threats and anxiety by fostering perspective-taking and empathy. However, as pointed-out by Pettigrew and Tropp (2008), such benefit of intergroup contact can backfire if it is not supported with common goals. Practically, a common goal-driven intergroup contact could be actualized through endorsing an inclusive, open international policy with which Muslim countries and the West are actively involved in mutual cooperation. This cooperation aims at achieving common goals in either technical domains such as water management, global warming, and health (Nadler & Shnabel, 2008) or general domains such as student exchanges and cultural exhibitions (Mashuri, Zaduqisti, & Supriyono, 2012).

## Figures and Tables

**Figure A.4:**
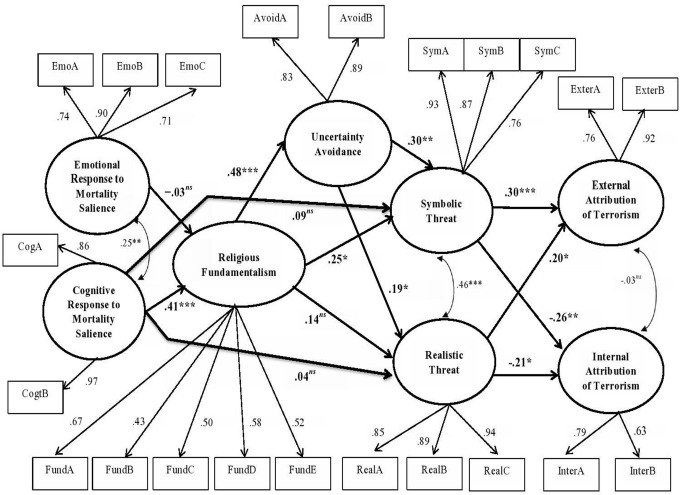
The Alternative Model 3 in which cognitive response to mortality salience is predicted as not only indirectly affecting symbolic threat and realistic threat via religious fundamentalism but also directly affecting each of these intergroup threats. *Note.* The numbers were standardized path coefficients and factor-loadings. ExterA−ExterB = External Attribution of Terrorism Parcel A and Parcel B; InterA−InterB = Internal Attribution of Terrorism Parcel A and Parcel B; SymA−SymC = Symbolic Threat Parcel A, parcel B, and Parcel C; RealA−RealC = Realistic Threat Parcel A, parcel B, and Parcel C; AvoidA−AvoidB = Uncertainty Avoidance Parcel A and Parcel B; FundA−FundE = Religious Fundamentalism Parcel A, Parcel B, Parcel C, Parcel D, and Parcel E; EmoA−EmoC = Emotional Response to Mortality Salience Parcel A, Parcel B, and Parcel C. CogA-CogB = Cognitive Response to Mortality Salience Parcel A and Parcel B. *^ns^p* ≥ .05 (not significant). *p < .05. **p < .01. ***p < .001. All factor-loadings were significant at *p* < .001.

## Appendix 1: A Complete List of Items Wording

### Emotional and Cognitive Responses to Mortality Salience

“Please imagine that your own death arouses in you” and “To what extent do you agree that as you physically die and once you are physically dead, you will feel emotions and think about the topics as the following?”

1. Sadness.
2. Fear.
3. Loneliness.
4. Anxiety.
5. Depression.
6. Heaven and Hell.
7. Physical decay.
8. Allah.
9. Religion.
10. Sin.

### Islamic Fundamentalism

1. Allah has given mankind a complete, unfailing guide to happiness and salvation, which must be totally followed.
2. All of the religions including Islam in the world have flaws and wrong teachings (R).
3. Of all the people on this earth, one group has a special relationship with Allah because it believes the most in HIS revealed truths and tries the hardest to follow HIS laws.
4. The long-established traditions in Islam show the best way to honor and serve Allah, and should never be compromised.
5. Islam must admit all its past failings, and adapt to modern life if it is to benefit humanity (R).
6. When you get right down to it, there are only two kinds of people in the world: the Righteous, who will be rewarded by Allah and the rest, who will not.
7. Islam has different versions of the truth, and may be equally right in their own way (R).
8. The basic cause of evil in this world is Satan, who is still constantly and ferociously fighting against Allah.
9. It is more important to be a good person than to believe in Allah and the right Islam (R).
10. No one religion including Islam is especially close to Allah, nor does Allah favour any particular group of believers (R).
11. Allah will punish most severely those who abandon his true Islam.
12. No single book of religious writings including Qur’an contains all the important truths about life (R).
13. It is silly to think people can be divided into "the Good" and "the Evil." Everyone does some good, and some bad things (R).
14. Allah's true followers must remember that HE requires them to constantly fight Satan and Satan's allies on this earth.
15. Parents should encourage their children to study all religions not just Islam without bias, than make up their own minds about what to believe (R).
16. Islam is a religion on this earth that teaches, without error, Allah's truth.
17. "Satan" is just the name people give to their own bad impulses. There really is *no such thing* as a diabolical "Prince of Darkness" who tempts us (R).
18. Whenever science and sacred Qur’an conflict, science must be wrong.
19. There is *no* body of teachings, or set of scriptures, including Qur’an, which is completely without error (R).
20. To lead the best, most meaningful life, one must belong to the one, true Islam.

### Uncertainty Avoidance

1. It is important to have instructions spelled out in detail so that I always know what I am expected to do.
2. It is important to closely follow instructions and procedures.
3. Rules and regulations are important because they inform me _of what is expected of me_
4. Standardized work procedures are helpful.
5. Instructions for operations are important.

### Symbolic Threat

1. I am very concerned about the Western ways of life that that are not in line with Moslem ways of life.
2. I worry at the West’s ways of life that contradict to Muslim’s ways of life.
3. The possibility that the values ​​and culture of the West will continue to displace and weaken the values ​​and culture of Islam is something that deeply worries me.
4. I think that the views or ideas of the Western world are very alarming because they deviate from the views and thoughts of Islam.
5. How the West interpret and judge the life I think is not in tune with how Muslims do, thereby putting the values ​​and culture of the West as a guide is something worrying.
6. I fear that in the current open society era, the influence of the values ​​and norms of the West will compete with and even displace the values ​​and norms of Islam.

### Realistic Threat

1. I fear that the economy of the Western world that is more developed and stronger than that of Islamic world will weaken the influence of the Islamic world in interstate competition.
2. I fear that the advancement of science and technology of the Western world that significantly surpasses that of the Islamic world will make the Western world become more powerful.
3. I fear that the more powerful and influential position of the Western world than that of Islamic world will weaken the power of Islamic world in interstate competition.
4. I fear that the Western world with advanced economy and technology will intervene to manage natural resources in the Islamic world so that the resources cannot be fully to increase the welfare of the Muslim.
5. I fear that the strong powers of the Western world will debilitate a bargaining position the Islamic world in the global competition.
6. I fear that the strong power of the Western world will make them easily intervening Islamic world’s domestic affairs and problems.

### External Attribution of Terrorism

1. Terrorism in Indonesia is created by the conspiracies of the West.
2. Terrorism in Indonesia is a term popularized by the West as a scapegoat to blame Muslims for the action they never did.
3. Terrorism in Indonesia is due to the West’s intervention to this country’s domestic problems.
4. Terrorism in Indonesia is clandestinely designed by the West’s intelligence.
5. Terrorism in Indonesia is engineered by the West to debilitate the image of Islam.
6. The West is the true perpetrator of terrorism in Indonesia.

### Internal Attribution of Terrorism

1. Terrorism in Indonesia is due to the errors radical Muslims have done in interpreting Islam.
2. Radical Islamists are the true perpetrator of terrorism in Indonesia.
3. Terrorism in Indonesia is attributable to radical Muslims’ misinterpretation of the meaning of Jihad.
4. Terrorism in Indonesia originates from radical Muslims’ narrow-mindedness in viewing and understanding the West.
